# Social inequality in working life expectancy in Sweden

**DOI:** 10.1007/s00391-018-01474-3

**Published:** 2018-11-09

**Authors:** Roland Kadefors, Kerstin Nilsson, Per-Olof Östergren, Lars Rylander, Maria Albin

**Affiliations:** 1 Department of Sociology and Work Science, University of Gothenburg, Box 705, 405 30 Gothenburg, Sweden; 2 Division of Occupational and Environmental Medicine, Lund University, Lund, Sweden; 3 Division of Social Medicine and Global Health, Lund University, Lund, Sweden; 4 Institute of Environmental Medicine, Karolinska Institutet, Stockholm, Sweden

**Keywords:** Retirement, Socioeconomic class, Disability pension, Health, Ancestry, Ruhestand, Sozioökonomische Klasse, Erwerbsunfähigkeitsrente, Gesundheit, Herkunft

## Abstract

**Background:**

In Sweden there is a socioeconomic divide between white and blue collar workers with respect to the risk for premature exit from working life. Disability pension has long represented a major reason behind early exits.

**Objective:**

The present investigation aimed at studying the effect on socioeconomic groups of new guidelines issued by the Swedish government in 2006, limiting the possibilities for applicants to be granted pension on medical grounds.

**Material and method:**

The study was based on register data comprising the prevalence of disability pension and premature age pension in different occupations in the age group 55–64 years, comparing the years 2006 and 2011.

**Results:**

It was found that in 2011 under the new guidelines, newly approved disability pensions had dropped by 70%. Women were affected more than men. The drop in disability pensions affected applicants within the two most prevalent diagnosis groups, mental disorders (a drop by 58%) and musculoskeletal disorders (a drop by 87%). In the same time period, the percentage in the age range 55–64 years choosing premature age pension more than doubled. An increase in the number of premature age pensions was more common in blue collar occupational groups than in white collar workers. Occupation had a higher impact on working life expectancy than country of birth.

**Conclusion:**

There are strong indications that many applicants, particularly blue collar workers, who had been unable to be granted disability pension under the new operational guidelines, instead choose premature retirement, a costly alternative for many individuals with already low pension benefits. The results indicate a tendency of passing on the societal costs of early labor market exits to various economic compensation arrangements, as well as to the individuals themselves.

## Zusammenfassung

**Hintergrund:**

In Schweden besteht eine sozioökonomische Disparität zwischen Arbeitern und Angestellten im Hinblick auf das Risiko eines vorzeitigen Ausscheidens aus dem Arbeitsleben. Die Erwerbsunfähigkeitsrente ist lange ein wesentlicher Grund für das vorzeitige Ausscheiden gewesen.

**Zielsetzung:**

Untersucht werden sollten die Auswirkungen neuer Leitlinien der schwedischen Regierung aus dem Jahr 2006, welche die Möglichkeiten, aus medizinischen Gründen berentet zu werden, einschränken.

**Material und Methoden:**

Die Studie basierte auf Registerdaten, anhand derer sich die Jahre 2006 und 2011 hinsichtlich der Prävalenz von Erwerbsunfähigkeits- und vorzeitiger Altersberentung für in verschiedenen Berufen Tätige im Alter zwischen 55 und 64 Jahren vergleichen ließ.

**Ergebnisse:**

Festgestellt wurde, dass im Jahr 2011 unter den veränderten Leitlinien die Anzahl neu genehmigter Erwerbsunfähigkeitsrenten um 70 % gesunken war. Frauen waren stärker betroffen als Männer. Der Rückgang der Erwerbsunfähigkeitsrenten betraf Antragstellende innerhalb der beiden häufigsten Diagnosegruppen: psychische und muskuloskelettale Erkrankungen (Rückgang um 58 bzw. 87 %). Im gleichen Zeitraum hatte sich der Prozentsatz der Beschäftigten, der sich für die vorzeitige Altersrente entschieden hatte, mehr als verdoppelt. Mehr Arbeiter als Angestellte hatten sich für eine vorzeitige Altersberentung entschieden. Der Beruf hatte höheren Einfluss auf die Erwerbslebensdauer als das Geburtsland.

**Fazit:**

Es gibt deutliche Anhaltspunkte dafür, dass viele Antragstellende, insbesondere Arbeiter, die nach den neuen Leitlinien keine Erwerbsunfähigkeitsrente erhalten konnten, stattdessen auf den vorzeitigen Ruhestand setzen, für viele Menschen mit absehbar niedrigen Rentenleistungen eine mit finanziellen Einbußen verbundene Alternative. Diese Ergebnisse deuten auf eine Tendenz hin, die gesellschaftlichen Kosten eines frühen Ausscheidens aus dem Arbeitsmarkt in unterschiedliche ökonomische Entschädigungsvereinbarungen einfließen zu lassen und an die Betroffenen selbst weiterzugeben.

## Introduction

Working life participation in the older population is high in Sweden compared to other EU countries; according to the Organisation for Economic Co-operation and Development (OECD) statistics in 2017 for 55–64-year-old women it reached 74.5% and for men 77.7% [1]. Nevertheless, there is political agreement that Swedes need to work more years in order to help sustain the old age pension system. Economic incentives aim at movement of the retirement norm^1^, currently 65 years of age, upwards [2]. A governmental bill to be introduced in the Swedish Parliament [3] includes specific suggestions to raise the age limit referred to in the Employment Protection Act from 67 to 69 years, and to change the earliest age at which people are entitled to draw their old-age pension from 61 to 64 years. Full pension benefits are now available at age 65 years; this age limit is to be adjusted upwards following the expected increased longevity. These changes will be implemented gradually and completed by 2026. The proposed changes in Sweden reflect an international trend. Approximately half of the OECD countries are introducing legislation that will increase the retirement age, with links to life expectancy in Denmark, Finland, Italy, the Netherlands, Portugal and the Slovak Republic. Across the OECD, the normal retirement age is expected to increase by 1.5 years for men and 2.1 years for women by 2060 [4]. Sweden does not have a fixed pension age. Since the pension system in built on life income, working more years will add significantly to pension benefits. In addition to the state pension, approximately 90% of Swedish employees have access to an employment pension, which is based on collective agreements between social partners.

Relying on economic incentives in order to bring about a longer working life in large parts of the labor market is built on the presumption that individuals have a free choice at what age they may retire; however, prospective retirees do not form a uniform group; they may be classified into four categories with respect to working into older age: (a) those who can and want to; (b) those who can but do not want to; (c) those who cannot but would like to, and (d) those who cannot, and do not want to [5,6,7]. Financial incentives to work could be attractive for those whose work ability is unaffected, for instance by health constraints. Therefore, such incentives may be relevant for individuals in the aforementioned categories (a) and (b), but less so for individuals in categories (c) and (d).

In a previous population study [8], this group showed that in the time period 2006–2011 the average working life exit age had moved upwards in almost all occupations, but that it differed between socioeconomic groups in both genders: “lost years” was defined as the number of years that people in a given occupation are expected to exit from the labor market before reaching age 65 years. Those in blue collar occupations^2^ exited working life earlier than those in almost all white collar occupations; there was a striking socioeconomic divide between blue and white collar workers in this respect. The difference between extremes in lost years before age 65 years (SSYK^3^ 919 “Other sales and services elementary occupations” versus SSYK 231 “College, university and higher education teaching professionals”) in 2011 amounted to approximately 4.7 years in men and 5.6 years in women.

An important question is what effects financial incentives may have in different socioeconomic groups. It is well known from studies in different economies that low-income groups tend to retire prematurely, even though they may face low pension benefits (e. g. [9,10]); however, socioeconomic determinants behind the decision to retire are manifold. Retirement norms may develop in different socioeconomic strata [11] and it has been shown that ill health may increase the likelihood of labor force exit into unemployment, disability pension and early retirement. Workers with low socioeconomic status, even after adjusting for ill health, have been found to be more likely to leave the labor force due to unemployment, disability pension and economic inactivity [12].

Even though work participation among older people is affected by many factors it is generally agreed that there is a strong influence of health on the age and the circumstances under which people retire. Those with health impairments have a heightened risk of prematurely dropping out of the labor force due to disability, unemployment or early retirement (e. g. [13,14,15]). It is also well known that life expectancy as well as perceived health has a socioeconomic gradient. This gradient, as defined by educational level or by income percentile, has increased for life expectancy in Sweden during the last decades, as it has in other western European countries, while the socioeconomic gradient regarding self-reported health remained fairly constant over time. The match between work demands and individual capacity is an important determinant for premature exit from the labor market. In a public health survey conducted in the Stockholm area in Sweden, 18% of the women and 20% of the men with only primary education stated that the physical work demands exceeded their capacity. The corresponding proportions for those with at least 3 years of university/college were 8% and 3% respectively [16]. This gradient may result from an excessive physical workload being most common in blue collar occupations, and a higher prevalence of concomitant disease reducing the work capacity in lower socioeconomic strata. Physical workload is the workplace exposure that contributes most to socioeconomic inequalities in health (e. g. [17]). Musculoskeletal disorders contribute over 50% of the excess in disability retirement between manual workers and high-level non-manual workers, both among men and women [18].

Disability pension, which may be granted to applicants in the age range 30–64 years, has long been a common source of economic support preceding retirement. For instance, in 2009 out of those who retired at age 65 years 40.9% of the women and 30.1% of the men had been on disability pension the year before they retired [19]; however, in 2006 the Swedish government issued new guidelines limiting the opportunities for applicants to be granted disability pension on medical grounds. The results were striking: more than 530,000 persons were on disability pension in 2006 compared to approximately 373,000 in 2011 [20]. In a previous study it was found that following the changes in the disability pension regulations, the proportion of individuals who had chosen early age pension or employment pension increased, especially among those with a low level of education [21]. Early age pension can be drawn at free will by a person who has reached an age of 61 years; at age 65 years this temporary status is converted to regular age pension.

The present study aimed at identification of the socioeconomic effects of the policy changes instituted in 2006, by mapping the development of approved disability pension applications within different diagnosis groups, with respect to gender and occupational background of applicants. The study further analyzed the proportion of individuals in different occupations and with different socioeconomic characteristics who had chosen early age pension. In addition to socioeconomic groups identified by occupation, attention was also paid to the effect of country of birth on working life exits.^4^

## Method and material

### Disability pension

This investigation aimed at analyzing the development of approved applications for disability pension in the time period 2006–2011, following the changes instituted in 2006. The study was based on register data comprising the prevalence in the age group 55–64 years of disability pension and early age pension in different occupations. Special emphasis was on the differences between women and men. Swedish Social Insurance Agency data pooled with LISA^5^ statistics [19,22] concerning approved disability pensions in 2006–2007 among persons in the age range 55–64 years were further analyzed with respect to socioeconomic patterns in the 10 occupations with the highest percentage of approved cases. This analysis was carried out using the 4‑digit SSYK code, based on the International Standard Classification of Occupations (ISCO-88) [23]. The classification between blue and white collar occupations was undertaken using a proposed translation key developed by Statistics Sweden [24], based on a set of criteria including nature of work, required length of education, and management responsibilities. It has been recognized that identification of socioeconomic status may take into account a person’s main type of activity, occupation, occupational status (self-employed/employee) and be divided into upper non-manual employees, lower non-manual employees, manual workers and self-employed [25].

### Early age pension

This population study aimed at an assessment of the proportion of individuals in different socioeconomic groups who had chosen early age pension in 2004 and in 2011. It was based on the Swedish national labor statistics covering all employees who had an occupational definition and who were 55–64 years of age in 2004 or in 2011. The individuals included in the study were classified in the age bracket 52–61 years in 2001 for the outcome age 55–64 years in 2004 and in 2011. In all, approximately 773,000 individuals were included in the study population for 2004, and 788,000 for 2011.

Occupations were identified according to the 3‑digit SSYK code, with special consideration paid to differences between males and females. In the data processing the focus was on the largest occupational groups; hence, the 50 most common occupations in men and women were analyzed out of the 112 occupations listed according to SSYK 3. The group sizes in the study are large with at least 1000 observations in each group.

### Working life exit and ancestry

In a previous report [8], the working life durations in different occupational groups were analyzed. In the present context, the same methodology was applied to account for the country of birth. The analysis was carried out as a population study employing methodology used in demographics to predict life expectancy at birth. Calculation of life expectancy in population statistics is based on data concerning the number of deaths in different age groups. Likewise, calculations of expected remaining working life duration are based on data of the exits from working life. These calculations comprise death, disability pension and long-term sick leave preceding disability pension. The study was based on the Swedish national labour statistics, covering all employees who had an occupational definition in November 2006. Another inclusion criterion was to be in the age 35–64 years during the measurement period 2007–2010. Exiting before age 65 years means that a person faces lost years in working life. At age 35 years, there remains 29.5 years for an individual to reach 65 years, the Swedish pension norm. Hence, the number of lost years is 29.5 years minus the expected number of years entailed in the analysis. For a more in-depth description of the method employed, see [8]. In the presentation of the ancestry statistics, the socioeconomic identification key based on occupation is applied again [24], but in this case SSYK 1, the highest aggregation level; this is to ascertain relevant group sizes.

## Results

### Disability pension

**Fig. 1:**
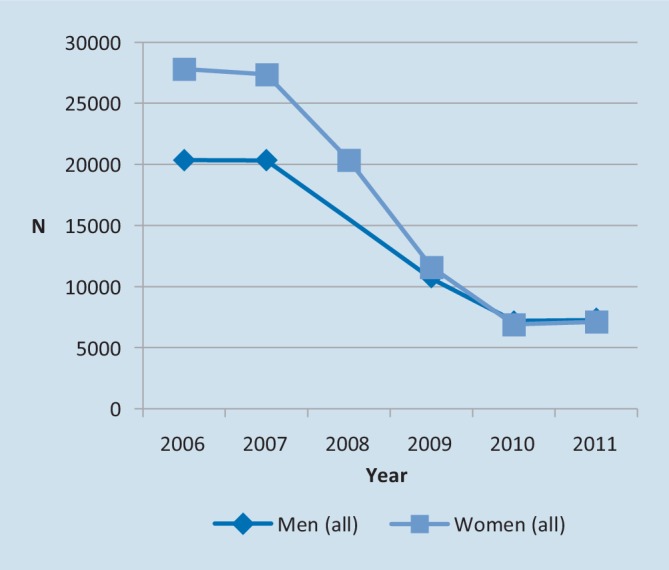
The number of approved applications for disability pension in women and men following the policy change in 2006. (Data source: Swedish Social Insurance Agency)

The effect of the policy change in 2006 was dramatic. In the time period 2006–2011, the total number of approved applications annually was reduced progressively from 48,176 to 14,369, i.e. by 70.0%. For women, the reduction was 74.4% and for men 64.3% (Fig. 1). Within the two major diagnostic groups according to the International Classification of Diseases (ICD-10-SE); F00–F99 (mental disorders) and M00–M99 (musculoskeletal illnesses), the reduction amounted to 58% and 87% respectively. In women the reductions amounted to 64.9% for mental disorders, and 91.1% for musculoskeletal disorders; in men the corresponding reductions were 48.8 and 90.0%, respectively.

**Fig. 2:**
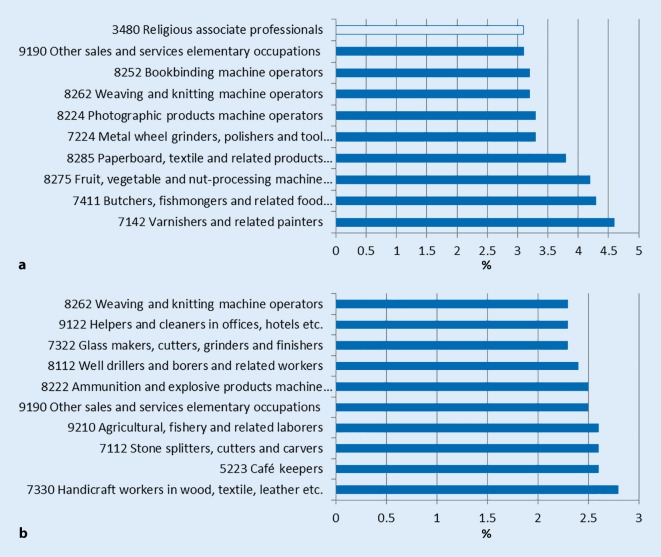
**a,** **b** The 10 occupations among Swedish women and men in the age range 50–64 years with the highest percentages of approved disability pensions before the policy change in 2006. Figures represent percentages of the total number of persons in the given age range, in the respective occupations. Note the different scales in **a** and **b**. The only occupation to be identified as white collar in these statistics is in **a** SSYK4 3480: Religious associate professionals. *White bars* white collar, *blue bars* blue collar. (Data sources: Sjögren Lindqvist [19] and Statistics Sweden)

Who were the people who had been granted disability pension before the policy change? Fig. 2a, b shows the 10 occupations with the highest percentage of the population sample in the age span 50–64 years. The mean percentages in women and men were 3.61% and 2.49% (*p* < 0.001), respectively, showing that it was significantly more common among women than men to be on disability pension before the change in governmental policies. It was found that among men, all 10 of the occupations singled out were characterized as blue collar, and among women 9 out of 10. In fact, extending the figures by another 10 high risk occupations also entails solely more new blue collar occupations for men and women.

### Early age pension

Table 1 shows the early pension prevalence in 2004 and 2011, and probabilities for the differences between blue and white collar occupations as evaluated by t‑tests. In 2004, there was no significant difference among women between white and blue collar workers in the most common occupations; however, it was more common among men in white collar occupations than in blue collar occupations to have chosen early age pension. In 2011, this difference had disappeared but among women blue collar now showed significantly higher percentages than white collar occupations.

**Table 1:** Prevalence of early age pension in socioeconomic groups in 2004 and 2011 (55–64-year-olds)

	2004			2011		
	White collar	Blue collar	Difference blue-white	White collar	Blue collar	Difference blue-white
Women	1.27 *N* = 30	1.93 *N* = 16	0.66 *p* > 0.05	4.57 *N* = 31	6.03 *N* = 14	1.46 *p* < 0.01
Men	2.69 *N* = 30	2.02 *N* = 32	−0.67 *p* < 0.01	6.01 *N* = 33	6.32 *N* = 35	0.31 *p* > 0.05

*N* the number of occupational groups identified as white or blue collar, where the number of individuals exceeded 1000

The prevalences of early age pension in the age group 55–64 years were compared in 2004 and 2011, before and after the change in disability pension policies, in the 50 most common occupations. What possible effects of the restrictions could be found in the early age pension statistics? Table 2 shows that the prevalence of persons in the age range 55–64 years who were on early age pension in 2004 and 2011 had increased, for men and women alike. The increase amounted to 30,564 persons.

**Table 2:** Early age pension prevalence and numbers in the age span 55–64 years in 2004 and 2011

	2004 (%)	2011 (%)	Increase (%)	Increase number
Women	1.7	5.5	3.8	15,564
Men	2.5	6.4	3.9	15,000

**Fig. 3:**
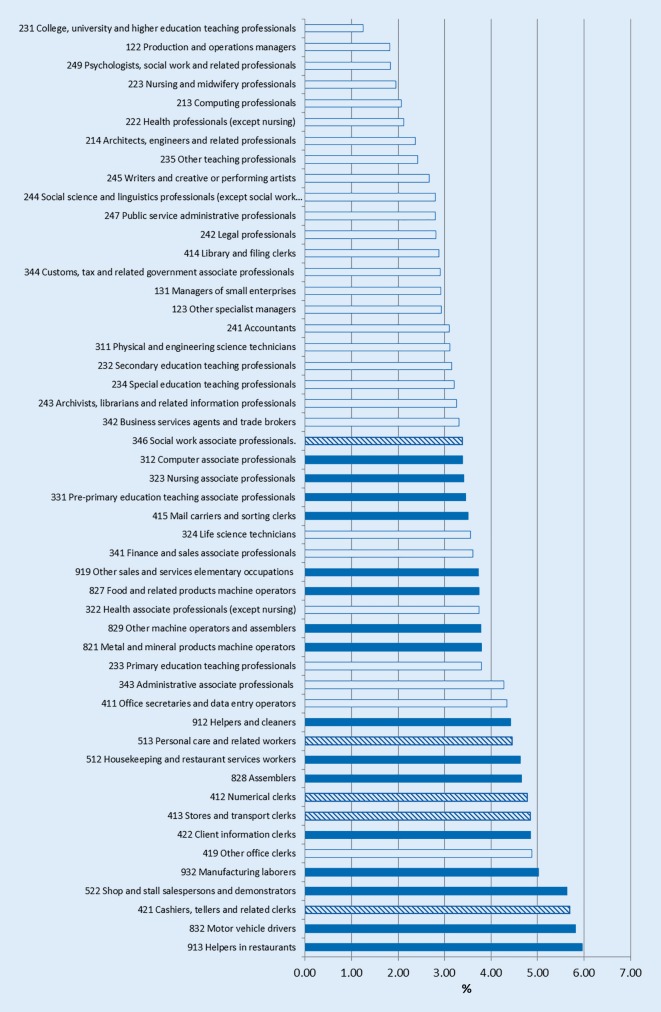
The change in age pension percentages 2006–2011 among Swedish women in the 50 most common occupational groups, also applying the socioeconomic criteria identifying occupations at the SSYK3 level. *White bars* white collar*, blue bars* blue collar, *striped bars* mixed blue and white collar. (Data source: Statistics Sweden)

**Fig. 4:**
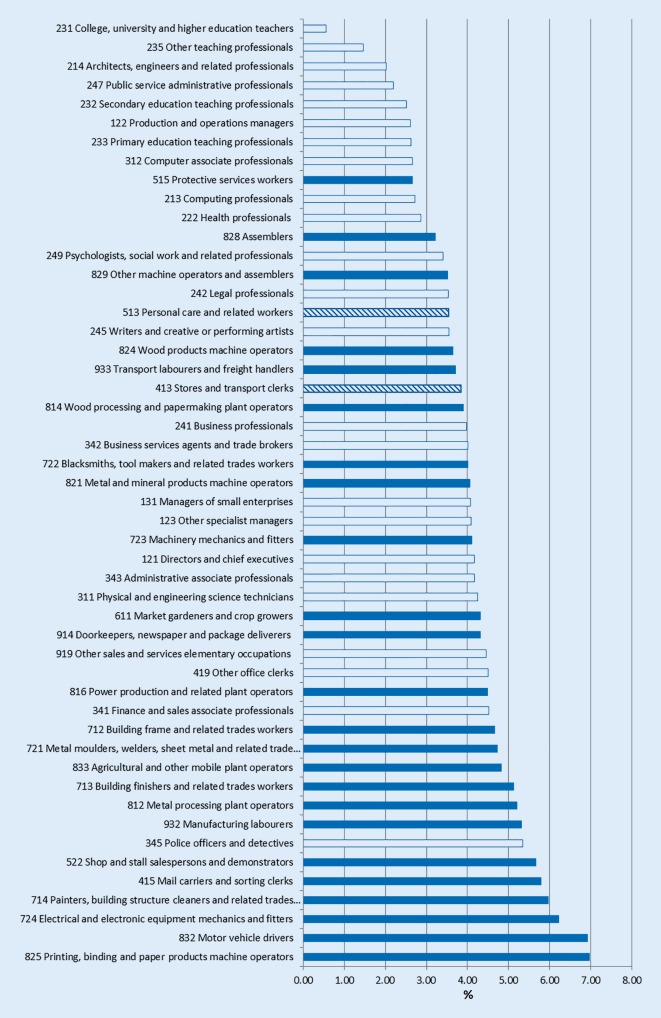
The change in age pension percentages 2006–2011 among Swedish men in the 50 most common occupational groups, also applying the socioeconomic criteria identifying occupations at the SSYK3 level. *White bars* white collar*, blue bars*: blue collar, *striped bars* mixed blue and white collar. (Data source: Statistics Sweden)

The analysis of the change in different occupations showed that in all 50 occupations studied in men and women, early age pension was more common in 2011 than in 2006. Figs. 3 and 4 show the change in age pension prevalence among women and men, also applying the socioeconomic criteria identifying occupations as blue or white collar. For instance, for the two extremes in Fig. 3 (SSYK 231 and SSYK 913), the prevalence of early age pension in women in 2006 was 0.96% and 2.05%, respectively and in 2011 the corresponding numbers were 2.21% and 8.00%. This means that for SSYK 913 (helpers in restaurants) there was an increase of 5.96%; for SSYK 231 (college, university and higher education teaching professionals) the prevalence increase was a mere 1.24%.

Some occupations were mixed blue and white collar; this means that according to the translation key [24], an SSYK 3 occupation may contain both blue and white collar SSYK 4 occupations. In women, out of the 50 occupations analyzed, the 10 occupations with the lowest increase in percentage change were all white collar, whereas out of the 10 most affected occupations 9 were blue collar or mixed (mean values of blue and white collar occupations: 6.03 and 4.57, respectively; *p* < 0.01).

Among men, 9 out of the 10 with the smallest changes were white collar and 1 was blue collar. Out of the occupations with the highest changes, 9 were blue, and 1 white collar (mean values of blue and white collar occupations: 4.42 and 3.19, respectively; *p* < 0.01).

### Working life exit and ancestry

Tables 3 and 4 show average lost years in working life before age 65 years in groups according to occupation^6^ and geographical area of birth. It is seen that the socioeconomic divide prevails; the number of lost years is higher among blue collars in all groups, irrespective of land of birth. In all occupational groups, the Swedish born exit work later than those of different ancestry, but there is no consistent pattern with respect to geographical areas outside Sweden.

**Table 3:** Lost years in working life before age 65 years according to ancestry (land of birth) and occupation in 2011 (women). (Data source: Statistics Sweden)

SSYK 1	Blue/white collar	Sweden	Nordic	Europe	Outside Europe
Legislators, senior officials and managers	White	0.39	0.56	0.85	0.46
Professionals	White	0.47	0.52	0.67	0.65
Technicians and associate professionals	Mixed	0.62	1.12	0.86	1.24
Clerks	Mixed	0.89	1.16	1.00	1.62
Service workers and shop sales workers	Blue	1.30	1.62	1.93	1.74
Craft and related trades workers	Blue	1.28	1.80	2.48	1.73
Plant and machine operators and assemblers	Blue	1.59	2.49	3.56	2.61
Elementary occupations	Blue	2.40	3.06	3.59	2.51

**Table 4:** Lost years in working life before age 65 years according to ancestry (land of birth) and occupation in 2011 (men). (Data source: Statistics Sweden)

Occupational group	Blue/white collar	Sweden	Nordic	Europe	Outside Europe
Legislators, senior officials and managers	White	0.38	0.92	0.39	0.69
Professionals	White	0.49	0.53	0.69	0.65
Technicians and associate professionals	Mixed	0.59	1.09	0.90	0.98
Clerks	Mixed	1.40	1.99	1.82	1.35
Service workers and shop sales workers	Blue	1.38	2.59	1.78	1.58
Craft and related trades workers	Blue	0.98	1.69	2.09	1.39
Plant and machine operators and assemblers	Blue	1.27	1.94	2.76	1.55
Elementary occupations	Blue	2.71	3.04	3.39	2.38

## Discussion

### Disability pension

The result of the study showed that the change in disability pension policies in 2006 affected blue collars more than white collars, and women more than men. These findings comply in part with the results in earlier studies [19,21], noting that many of the most affected occupations before the change could be characterized as manual jobs with low requirements of formal education. These characteristics are part of the criteria applied by Statistics Sweden [24] in the development of a key linking SSYK code to socioeconomic group. In the present analysis, these affected occupations were classified as blue collar.

**Fig. 5:**
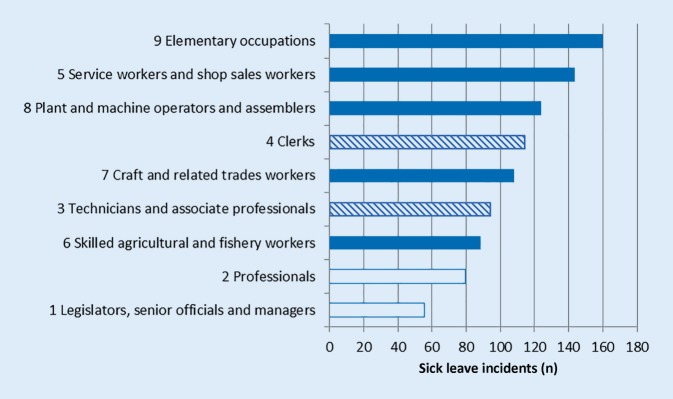
The number of sick leave incidents per 1000 employees in 2006 in different occupational groups according to SSYK 1, for women and men combined. *White bars* white collars, *blue bars* blue collars, *striped bars* mixed. (Data source: Swedish Social Insurance Agency)

The high occurrence among blue collar workers when it comes to accepted applications for disability pension before policy change complies with statistics from the Swedish Social Insurance Agency with respect to sick leave incidence [20]. Fig. 5 shows the numbers of sick leave per 1000 employees for the year 2006 in different occupations, classified according to SSYK 1. It is clear that the same distinction gradient between blue and white collar jobs exists with respect to sick leave as well as concerning disability and early age pension, further illustrating previous conclusions with respect to the importance of social determinants on health and retirement generally [26,27].

### Early age pension

In the present study it was found that following the 2006 change in disability pension policies, in 2011 there was a significant increase in the number of older persons who had chosen to take early age pension. This development concerned blue collars more than white collars, and women more than men.

For an older person who has been denied disability pension on medical grounds, there are a limited number of possible options, including continued work despite poor health, unemployment, sick leave, relying on relatives, or, as a last resort, social security. In this situation, it is understandable that early age pension may be seen as a solution, even though taking retirement pension before age 65 years negatively affects lifelong pension benefits. It is a costly alternative for most people, but for those who may have acquired few pension points (perhaps worked few years, part time or with low salary) it may be seen as an economically acceptable way out.

A closer analysis of the white collar data reveals that a particularly high prevalence of persons on early age pension was found among male managers, for instance SSYK 121, directors and chief executives, and SSYK 131, managers of small enterprises (5.1% and 5.2%, respectively, compared to 2.5% across all occupations). Among women, the corresponding figures were 2.7% and 2.1%, compared to 1.7% across all occupations. There seems to be a difference between male and female behavior in this respect.

### Working life exit and ancestry

It was found that the socioeconomic divide between blue and white collars exists across all groups irrespective of the country of origin. Those born outside Sweden tended to exit work earlier than Swedes. Based on statistics concerning disability pensions in 2006–2007 in groups of persons living in Sweden but with different ancestry, it has been reported that those who had immigrated to Sweden had a higher probability than the Swedish born to be on disability pension [19]; however, it was found that when controlling for occupation, the differences between groups of different origins decreased; among men, the probability of exiting due to disability pension became independent of country of birth, and also among women, the differences were markedly reduced. The results of this study comply largely with these findings; the occupation tended to have a larger influence on working life exits than ancestry.

### Sociodemographic differences and the policy change

The gender differences observed in the present report with respect to disability pension before the policy change in 2006 reflects that in Sweden, periods with long (>60 days sick leave) became more common among women than among men in the early 1980s, and the female to male ratio increased sharply up almost twofold from the early 1990s and onwards [28]. The Swedish labor market is highly gendered and the difference in female to male sick leave is largely driven by higher sickness absence in the occupations which are predominantly female (in healthcare and education). Women in the health sector reported the highest prevalence of low control in the Swedish Work Environment Survey in the early 1990s [29], and this structural difference prevails [30]. The results with respect to country of origin comply with statistics concerning sick leave in socioeconomic groups, showing that insured immigrants have a slightly higher risk of sickness benefit compared to Swedish born people. This difference was primarily explained by the fact that immigrants usually work in the service industry, healthcare and sales industry, which are occupations with relatively high levels of sickness absence [31]. Women had higher risk than men, particularly high among immigrants from regions suffering from armed conflicts.

## Conclusion

This study showed that the guidelines introduced by the Swedish government curtailing the possibilities for applicants to be granted disability pension affected women more than men, and blue collar more than white collar occupations. There was an apparent flow among those denied disability pension to early age pension, in particular in blue collars. The findings indicate that the budgetary savings of disability pensions arrived at by the government were at least to some extent transferred to the individual persons. The socioeconomic divide between white and blue collars prevailed also with respect to working life exits in groups of different ancestry. The restrictive terms for awarding disability pension, combined with the proposed higher age for full retirement benefits, and the strong economic benefits available to those who work longer, will increase the social inequalities. There is a need to take into account the effect on different socioeconomic groups of the changes currently being introduced in the welfare systems.

## Footnotes

1 “Norm” in the present context, is taken to signify the retirement behavior chosen by a majority of the eligible population.

2 Here we use the traditional concept “blue and white collars” rather than the perhaps more modern alternative “manual workers and non-manual employees”.

3 SSYK: Swedish Standard Classification of Occupations.

4 In the different parts of reporting, time periods vary to some extent. This depends on the availability of data, which differs depending on the data source.

5 LISA: Longitudinal integration database for health insurance and labour market studies. lisa@scb.se.

6 Note: SSYK 6 (Skilled agricultural and fishery workers) was left out due to low numbers in some cells.
